# The Care Coordinator’s Tasks During the Implementation of an Integrated Care Pathway for Older Patients: A Qualitative Study Based on the French National “Health Pathway of Seniors for Preserved Autonomy” Pilot Program

**DOI:** 10.5334/ijic.5977

**Published:** 2022-04-01

**Authors:** L. Douze, C. Di Martino, M. Calafiore, L. Averlant, Ch Peynot, M. Lotin, A. Delesalle, D. Dambre, M. Egot, A. Fabianek, M. M. Defebvre, C. Bugny, J. Thébault, F. Puisieux, S. Pelayo, J. B. Beuscart

**Affiliations:** 1 Inserm, CIC-IT 1403, F-59000 Lille, FR; 2 Univ. Lille, CHU Lille, ULR 2694 - METRICS : Évaluation des technologies de santé et des pratiques médicales, F-59000 Lille, FR; 3 Univ. Lille, Département de médecine générale, F-59000 Lille, FR; 4 Geriatrics Department, Lille Catholic Hospitals, University of Lille, Lomme, FR; 5 Centre Hospitalier de Saint-Amand-les-Eaux, Service de médecine polyvalente, FR; 6 Centre Hospitalier de Denain, Service de Pharmacie, FR; 7 Centre Hospitalier de Denain, Service de Gériatrie, FR; 8 ARS Hauts de France, FR; 9 Univ. Lille, ULR 4072 – PSITEC – Psychologie : Interactions, Temps, Émotions, Cognition, F-59000 Lille, FR

**Keywords:** older adults, frailty, care coordinator, integrated care, task analysis

## Abstract

**Background:**

Although integrated care and care coordination are known to be beneficial for older adults’ population, the specific tasks of a Care Coordinator (CC) for integrated care pathways for this population have not been studied in detail.

**Setting & Subjects:**

The French national pilot program PAERPA provided an integrated care pathway for older adults. In North France, a CC was recruited to support patients and professionals.

**Objectives:**

(i) To analyse the CC’s tasks in an integrated care pathway for older patients, and (ii) to record perceptions on the CC’s tasks among the participating general practitioners (GP) and community pharmacists.

**Design & Methods:**

Qualitative, two-phase study: (i) Task analysis of the CC’s tasks, to compare the planned and actual tasks; (ii) semi-structured interviews among GPs and community pharmacists involved in the pathway.

**Results:**

(i) The task analysis showed that the CC’s actual tasks differed from planned tasks. The CC was only meant to be involved in the early stages of the process; actually, the CC undertook more or even unforeseen tasks in coordination, communication, and administrative support throughout the care pathways. (ii) The 28 interviewed healthcare professionals considered the CC’s tasks to be essential to the success of pathways. They appreciated the CC’s administrative support. However, CC’s tasks related to interprofessional communication, and patient and family information, were controversially perceived among GPs and pharmacists.

**Conclusions:**

The CC’s tasks in an integrated care pathway for older adults showed that the CC’s overall workload was greater than expected and appreciated by healthcare professionals.

## Introduction

In France, the care of older adults is historically organized according to a professional model and become a succession of fragmented acts and procedures, managed by the patient or family, without full coordination between the various health care professionals or between hospital and community settings. The importance of an integrated care pathway and the strong need for a coordinator figure, as revealed by studies on the care of older adults living with frailty, seems to be precisely related to this important fragmentation of practices [1,2,3,4,5].

Kodner has defined integrated care as “a coherent set of methods and models on the funding, administrative, organizational, service delivery and clinical levels designed to create connectivity, alignment and collaboration within and between the cure and care sectors” [6]. The goal of these methods and models is to enhance quality of care and quality of life, consumer satisfaction and system efficiency for patients with complex, long term problems cutting across multiple services, providers, and settings. Valentin et al. [7] described three levels of integrated care frameworks: a macro (system) level, a meso level (professional, functional and organizational integration), and micro level (clinical/service integration).

Coordination is known to be a key factor in improving the quality of integrated care at all levels [2,8,9,10,11,12]. Researchers have extensively studied the modalities of care coordination and the benefits provided by care coordinators (CCs) in medical and/or social care settings [13,14,15,16]. Whereas quantitative studies of the effectiveness of care coordination have given conflicting results [11,17,18], qualitative studies show that the presence of a CC is generally perceived by the patients’ families and the participating healthcare professionals (HCPs) to be a factor that improves care [19]. Although it is clear that integrated care and care coordination are appropriate responses to the problem of caring for older adults living with frailty [1,20,21,22,23,24,25,26], most of the literature data on care coordination come from studies of patients (of all ages) with chronic illnesses [14,26,27,28,29,30].

Care coordination in integrated care for older adults living with frailty has been studied [23,24,25,26,27,28,29,30], but the professional figure of the care coordinator was under-researched. Some articles described him/her as a support for other health professionals, the person in charge of knowing the patient, the professional and the health system, and connecting them to each other [13,19].

In the analysis of care coordination for patients with chronic diseases, Kianfar et al. defined three categories of tasks, related to communication, relational coordination/relationship building and follow-up [28,29]. However, the specific tasks performed by the CC were poorly reported in the literature [31].

The specific tasks of a CC in an integrated care pathway for older people living with frailty have not been fully explored yet. No study has described the sharing and distribution of tasks between HCPs and CCs, nor has it compared planned and actual CC tasks. The “Health Pathway of Seniors for Preserved Autonomy” (PAERPA) French national experiment intended to provide a response to the problems arising from this fragmentation of care and lack of coordination in care of older adults living with frailty [32]. It fully meets Kodner’s definition and Valentin’s description of integrated care [6]. PAERPA was deployed in 16 areas of the country by the French Ministry of Social Affairs and Health between October 2014 and December 2019. It provided an integrated care pathway for older adults living with frailty (aged 75 and over) by coordinating the locally available medical and social care services. It provides older adults living with frailty with a personalized, integrated, multidisciplinary health plan. Both the HCPs and the patient had to sign up to the plan. A personal drug plan (focused on the risk of adverse drug events) had been developed in the Valenciennois-Quercitain area. The personal drug plan was initiated by a dedicated team during the patient’s hospital stay. Upon discharge, the personal drug plan continues in the community setting, and always includes at least one GP and one community pharmacist. In the Valenciennois-Quercitain area of northern France, the PAERPA program the recruitment of a CC. Although medical responsibility remained with each patient’s general practitioner (GP), the CC’s role was to inform and support HCPs and patients during the implementation of this new integrated care pathway.

Fragmentation of care and lack of coordination are not a problem exclusive to France, but it is recognized internationally and seems to affect in particular older adults living with frailty [31]. The presence of a care co-ordinator could respond to both issues. A detailed knowledge of the tasks he/she performs appears necessary.

This study based on PAERPA pilot program has two objectives: (i) analyse the CC’s tasks in an integrated care pathway for frail older patients, and (ii) record perceptions on the CC’s tasks among the participating GPs and community pharmacists involved in the care pathway.

## Method

### Design

**Figure 1:**
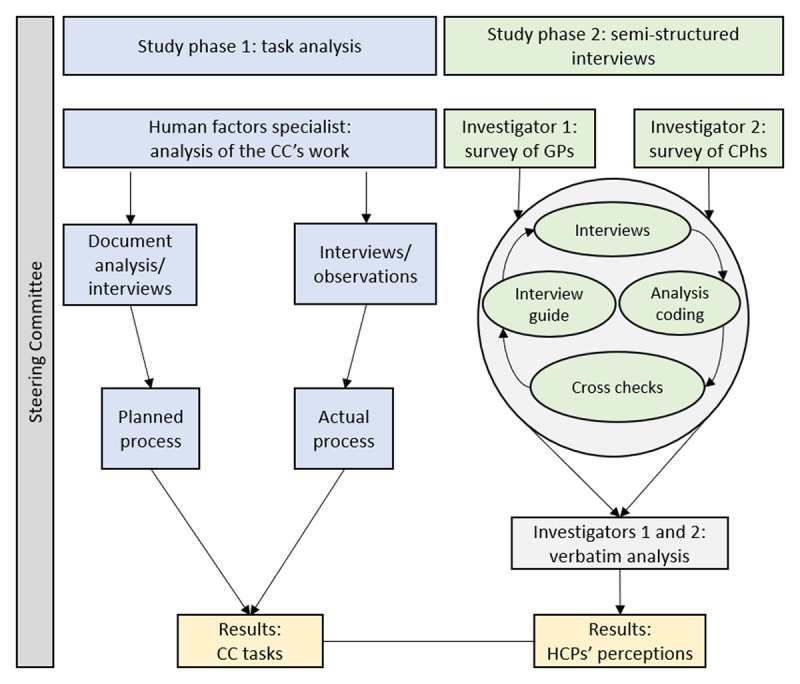
Methodology of the two complementary study phases (CC: care coordinator, GP: general practitioner; CPh: community pharmacist; HCP: healthcare professional).

We combined task analysis and semi-structured interviews in a two-phases qualitative assessment of the CC’s tasks as part of an integrated care pathway for older adults living with frailty. The two-phase study was carried out between December 2016 and December 2018 (***Figure 1***).

Phase 1: task analysis of the integrated care pathway initiated at Denain General Hospital (Denain, France) for the review of medication in older adults living with frailty. This analysis was carried out by a human factors’ specialist (CP). The task analysis focused on the tasks of the care coordinator, and on the interactions of the care coordinator with the other stakeholders of the pathways, during the whole duration of the pathways.

Phase 2: semi-structured interviews to gauge the perceptions of the GPs and community pharmacists having participated in the integrated care pathway. The two interviewers (ML and AD) worked separately but in a coordinated manner. Two other researchers (LD and CDM) synthesized the data on the CC’s tasks.

Both study phases were supervised by a steering committee (JBB, MC, LA, and SP). During monthly meetings with the investigators, the committee members advised on and validated the data collection and analysis methods, and discussed the results generated in each phase. The study’s results were reported in accordance with the Consolidated Criteria for Reporting Qualitative Research. Thirty-one of the 32 items on the COREQ checklist were completed (see the checklist provided as Suppl. Data 1).

### Ethical aspects

During the task analysis (phase 1), no personal or confidential data were collected, and no audio or video recordings were made. For the interviews (phase 2), the HCPs gave their written, informed consent to participation. The audio recordings were destroyed after transcription. As this type of study is not subject to the French legislation on clinical trials (government decree 2016–1537, dated November 16^th^, 2016), neither registration with the French National Data Protection Commission nor approval by an independent ethics committee was necessary [33].

### Phase 1: Task analysis

We assessed the planned and the actual CC tasks in integrated care for medication reviews among older adults living with frailty at Denain General Hospital. To this end, we combine a document analysis with ethnographic methods (interviews and observations).

Firstly, we analysed the local project’s implementation documents (e.g., action sheets), procedures, team activity reports, and tools (e.g., the person’s risk assessment grid).

Secondly, stakeholders in charge for conceiving and drafting the guidelines and work plan of the care coordinator were interviewed. The guide prepared for the semi-structured interviews could evolve and/or be adapted if necessary. Document analysis and interviews with stakeholders in charge for conceiving CC work plan enable us to identify CC planned tasks.

Thirdly, we carried out non-interventional observations of the actual tasks performed by the CC and any other stakeholder (especially GPs and community pharmacists), who interacted closely with the CC during the implementation of the integrated care pathway. When it was deemed necessary, they were questioned in order to have precision on their sharing actual tasks with the CC. Notes taken during and after observations served to identify the CC actual tasks evolution in its interaction with other HCPs.

At the time of the study, only one CC had been recruited. The CC was qualified in care management and gerontology.

The planned and actual CC tasks were then compared to highlight differences. All the results were validated by the Steering Committee. This study was carried out by a master’s degree student in human factors (CP), under the supervision of two human factors specialists (LD and SP). The investigator did not have prior relationships with any of the study participants or interviewees and introduced himself by explaining that the study was part of his master’s thesis project.

### Phase 2: semi-structured interviews

At the start of the project, there were 348 GPs and 148 community pharmacists in the Valenciennois-Quercitain area. All GPs and community pharmacists having participated in a personal drug plan initiated at Denain General Hospital were eligible for the present study. The list of HCPs having agreed or refused to participate in the pilot program was provided by the PAERPA support centre. The HCPs were contacted by phone, and those who agreed to participate were included in the study. Each of the two investigators (ML and AD) drafted an interview guide. One investigator (ML) performed all the interviews with the GPs, and the other (AD) performed all the interviews with the community pharmacists.

The interview guide invited professionals who were asked to participate in the integrated care pathway to express themselves freely about their experience. In agreement with the steering committee, this guide could be modified as the interview phase progressed. The interviews were conducted between March and July 2017 at the HCP’s office or pharmacy. Only the investigator and the interviewed HCP were present during the interview. The series of interviews continued until no new concepts emerged; this absence was checked by performing two additional interviews. Each interview was audio-recorded, transcribed manually and anonymously in its entirety, and then reported verbatim. The results were coded and analyzed by the respective interviewers according to the grounded theory approach, using Nvivo® software (QSR International Pty Ltd, Melbourne, Australia). The verbatim data were double-checked by the other interviewer.

The interviewers were house officers who had attended a standardized, two-day training course on qualitative research at the Lille Faculty of Medicine (Lille, France). They did not have prior relationships with any of the interviewed HCPs.

Finally, verbatims concerning the care coordinator were isolated from the other topics addressed by the participants and were reviewed by two other researchers (LD and CDM) one by one to relate them to the results of the task analysis.

## Results

### Analysis of the CC’s tasks in an integrated care pathway with medication reviews for older adults living with frailty

We performed four semi-structured interviews (total duration: 5 h) and seven non-interventional observations (total duration: 30 h). The durations of the observations and interviews are given in Suppl. Data 2. Our analysis focused on the CC’s activities. Other stakeholders (GP, community pharmacists) identified during the analyses were also interviewed or observed during their interactions with the CC.

We identified eight (8) steps in the process of implementing an integrated care pathway with a medication review for older adults living with frailty hospitalized patients (***Table 1***). Our analysis also provided a detailed list of planned vs. actual tasks completed in each step of the process. The results of this comparison are shown in Suppl. Data 3. The actual steps in the process did correspond to the planned steps, although there were differences in the various stakeholders’ level of involvement.

**Table 1:** Task analysis: stakeholder involvement at each step in the integrated care pathway.


STEP	PLANNED STAKEHOLDERS			ACTUAL STAKEHOLDERS		
	
	H	HCP	CC	H	HCP	CC

1. SET-UP	X	X	X	↗	=	=

2. MEDICATION REVIEW	X			=		

3. INITIATION OF A PERSONAL DRUG PLAN		X	(X)	↗	=	↗

4. LOCAL CLINICAL COORDINATION TEAM		X	(X)		=	↗

5. ASSESSMENTS		X			=	↗

6. SUMMARY		X			↘	↗

7. IMPLEMENTATION AND MONITORING		X			↘	↗

8. CLOSE-DOWN			X			↗


X: planned intervention by the stakeholder. (X): optional intervention by the stakeholder. ↗: unexpectedly involved in a task, or more involved in the task than planned. ↘: less involved in the task than planned. =: involved to the extent planned. H: hospital staff; HCP: healthcare professional; CC: care coordinator.

The overall workload was greater in the actual process than in the planned process. The comparative task analysis (Suppl. Data 3) shows that the number of actual tasks (between 23 and 37, depending on the step) was almost twice that planned. More specifically, the CC was involved in four times more tasks (n=24) than planned (n=6). All the CC’s optional tasks became permanent (steps 3 and 4). Many of the tasks intended for other stakeholders were taken on by the CC or required her additional involvement.

At first, the CC’s intended role was to train the HCPs with regard to the personal drug plan. The CC was necessarily involved in the first step, i.e., present the PAERPA project to the HCPs and ask them whether or not they wished to participate. The CC’s participation in steps 3 and 4 was optional, i.e. providing methodological support to the HCPs during their first personal drug plans or for complex cases, and helping the HCPs to set up the requisite systems and carry out the various actions. The CC was also involved in the last (8^th^) step, i.e. performing administrative work.

We found that after three years of the programme, the CC had become involved in 7 of the 8 steps. In steps 3 and 4, the CC made the patient’s discharge from hospital safer by smoothing out interactions between hospital staff and the community HCPs. The CC completed the information collected in hospital and sent it to the HCPs concerned (i.e., at least the patient’s GP and community pharmacist) so that the action plan could be initiated. In stages 3 to 6, the CC tended to take responsibility for all the tasks that did not require the expertise of a physician or other HCP. The CC took care of all the tasks related to administration, centralization of information, and operational organization of the actions (contacting the HCPs, summarizing the actions implemented, following up on the actions, etc.). Accordingly, the other stakeholders were less involved than expected in some of the other steps (mainly steps 6 and 7). The CC also had unplanned contact with the patient (notably to confirm his/her agreement to continuation of the personal drug plan, in step 4) and was alerted of changes in the patient’s status (in all steps).

In summary, the CC performed more administrative tasks than initially expected, and was more heavily involved in the planned tasks. The CC’s new tasks were mostly related to coordination and communication fields. In terms of coordination, the CC: (i) was in charge of operational organization of the personal drug plan by coordinating the actions and their follow-up; (ii) identified and contacted the stakeholders to be involved in the personal drug plan; (iii) aggregated or summarized information to be usable for different stakeholders at different steps of the pathway. In terms of communication, the CC was the privileged channel of communication between hospital and community settings, but also between healthcare professionals in community and patient. The CC ensured that the information was collected, updated, and transferred to the right stakeholder at the right time.

### Analysis of HCPs’ perceptions about the CC’s actual work in an integrated care pathway for medication review for older adults living with frailty

Overall, 53 GPs and 41 community pharmacists were eligible for inclusion in the study, and 35 GPs and 16 community pharmacists were contacted. Eighteen GPs and 10 community pharmacists agreed to participate in the study, and all 28 were interviewed.

The characteristics of the participating HCPs and the interview durations are summarized in Suppl. Data 2. The results of the two series of interviews are summarized in ***Table 2***. A list of the HCPs’ most significant statements about the CC is provided in Suppl. Data 2.

**Table 2:** A summary of the general practitioners and community pharmacists’ perceptions about the care coordinator’s task.


THE CARE COORDINATOR’S TASKS	COMMUNICATION	COORDINATION	ADMINISTRATION	OTHER TASKS

The general practitioners’ perceptions	Communication about the project: essential, needs to be reinforced Communication between the healthcare professionals: appreciated Communication about the patient (summary): to be reinforced	Overall coordination of actions: appreciated Conflicts of authority, according to some general practitioners	Administrative support: essential, appreciated Substantial time savings: much appreciated	The issue of physician-patient confidentiality was raised The lack of an IT platform was highlighted

The community pharmacists’ perceptions	Communication about the project: essential, needs to be reinforced Communication between the care coordinator and healthcare professionals: generally appreciated but hinders direct communication between community pharmacists and general practitioners. Meetings attended by all the healthcare professionals were wished for Communication about the patient (summary): to be reinforced.	Overall coordination of actions: appreciated Meetings attended by all the healthcare professionals were wished for	Administrative support: appreciated Substantial time savings: much appreciated	


The GPs’ and community pharmacists’ (CPh) perceptions of the CC’s tasks matched the tasks actually performed by the CC during the program.

1) The actual workload required for smooth operation of the process was too great for the HCPs alone. Delegating some tasks to the CC saved time and was unanimously considered to be essential for smooth operation of the process.

**GP2:** “We don’t have time to manage everything […], so the CC takes some of the load off us”; **GP6:** “The PAERPA process allows tasks to be delegated”; **CPh6:** “Well [without the CC], it wouldn’t be possible”; **GP18:** “[Interviewer]: Would you consider participation in the PAERPA without a CC? - No. She’s the lynchpin of the whole operation”.

2) The HCPs expressed the need for a single person who knows the process inside out and who can provide support them at all the stages.

**GP11:** “Of course… in all networked or similar systems, it’s good when there’s a person who really knows the system inside out, with all the tricks of the trade, the phone numbers, the administrative stuff that has to be done, and stuff like that. Otherwise, I reckon it’s a little bit overwhelming. It’s complicated”; **GP14** “The GP’s role is a bit like that of the pharmacist; you’re a stakeholder but you need to know what the others are doing. And there has to be a coordinator”.

3) The CC’s work activities were still perceived as especially being linked to the planned tasks, in terms of providing information during the project presentation phase and providing administrative support. All the HCPs were grateful for the CC’s greater involvement in administrative tasks.

**GP7:** “The support was valuable because we were discovering how the file was set up, and I think we needed this support”; **GP9:** “The CC is mainly there to provide information to physicians and other healthcare professionals”; **CPh7:** “The CC takes on some of the administrative burden”; **GP3:** “I don’t think that we have to fill out the papers any more,… it’s different from the start [of the program], so that’s good […]; we don’t get annoyed anymore because… it’s the coordinator who does all the administrative stuff that we had to do…”.

4) Some HCPs highlighted the CC’s lack of medical training. The problem of handling confidential medical information was also mentioned.

**GP7:** “Well, the CC is an administrator; she’s not from the medical professions, she’s not a healthcare professional” and “Physician-patient confidentially is also a problem; the CC is aware of the patient’s confidential medical information”. **GP14:** “I am not bothered by the fact that the CC is not a healthcare professional or is not highly qualified; she essentially provides us with administrative support”.

5) Some HCPs considered that the intensification and extension of the CC’s tasks were linked to the lack of adequate IT (information technology) support.

**CPh2** “It [the CC’s work] is essential! She’s the person that links the HCPs together - especially since we do not have any IT support”.

6) The extension of the CC’s involvement to coordination and communication tasks was controversial. Some GPs did not want the CC to be involved in coordination, whereas some community pharmacists were in favor of the CC’s facilitation of communication between HCPs. Other community pharmacists would have preferred more direct dialogue with GPs:

**GP15:** “At the outset, I expected the GP to be at the heart of the project. It ends up being almost everyone except the GP”; “I think the GP should be the coordinator, the referrer. We should do what we did before, and shouldn’t be put at the end of the chain - we have the impression that we have been pushed to the end of the chain”. **CPh4** “I think it’s good that there’s an intermediary because I can’t imagine the geriatrician calling us to tell us that so-and-so is about to go home. Maybe I’m wrong but it’s not yet common practice. But if there’s an intermediary, that’s fine with us”; **CPh9:** “Dialogue with the GPs is not happening… She [the CC] acts as the intermediary”.

## Discussion

The objective of this article was to present the analysis of care coordinator tasks in an integrated care pilot program for older adult living with frailty, focused on drug related problems. A task analysis of the care coordinator (CC) was performed, by comparing the planned process (defined before the beginning of the program) and the actual process (after 3 years of implementation), to identify the tasks and evolution of tasks of care coordinator. General practitioners (GP) and community pharmacists who take part of the program were interviewed to gather their perception about care coordinator tasks.

Several results can be highlighted. The workload was underestimated when integrated care pathway was created and could not be absorbed by the healthcare professionals (HCPs) of the patient. The CC could adapt his/her tasks to the constraints encountered during the implementation phases. This relieved the HCPs and was appreciated, but some HCPs had a feeling of loss of supervision over the process or the patient case. A reflection must be carried out to define the relationship with each professional, and an adaptability to each situation must be left to the care coordinator. Moreover, this study showed that the CC is not necessarily a health professional. The CC took on non-medical tasks that allowed HCPs to concentrate on tasks related to their medical and/or paramedical expertise. Finally, the details of the tasks performed by the care coordinator described in this article may help in the construction of other similar care pathways, with insights on the tasks and the workload that may be assigned to the different stakeholders.

Coordination is known to improve the quality of integrated care; in geriatric medicine, the value of integrated care is acknowledged because of the complexity of the medical and social issues [2,8,9,10,11,21,24]. Moreover, the high iatrogenic risk in this population means that medication review will become a core factor in patient management [34]. Hannigan et al. reported the ever-present dichotomy between “imagined” coordination and actual coordination [35]. Weaver et al. emphasized that each care coordination task must be viewed in terms of stakeholders’ professional and interprofessional practices [30].

Our results can be related to the work of Kianfar et al. [28,29], who determined three categories of activities, namely communication, relational coordination/relationship building, and monitoring. Communication refers, as in our study, to the transmission of information about the patient to the various team members. Monitoring includes the activities of supervising the process, identifying changes in the situation to be considered and anticipating their impact in the process. In our study, several tasks of the CC were related to monitoring, such as assessment, summary, or implementation and monitoring tasks. Relationship building refers to the construction of communication and trust between the care coordinator and patient and other stakeholders. It was not identified as a specific task in our study because it was associated with communication activities and was spread among other tasks. But it was an important part of care coordinator activity, especially during the implementation phases.

The literature on care coordination mainly covers projects coordinated by a nurse or another HCP, rather than the GP [8,11,14,15,18,19,29,36,37,38]. Al-Kaiyat et al. have shown that the nurse-coordinator’s role is evolving [37]. Furthermore, Parker et al. analyzed the relationship between the nurse’s coordination activities and the GP’s actual role [38]. These studies have shown that (i) the CC is often a nurse, (ii) there may be a conflict of authority between the nurse and the GP, and (iii) care coordination may be viewed simply as the provision of support to GPs [37,38]. Our study gave new insights into care coordination because we studied a CC who was not an HCP. Moreover, the links between the CC on one hand and the community pharmacists and GPs on the other were studied by analyzing the HCPs’ work and by conducting interviews on their perceptions. Our comparison of these two methodological approaches showed that the GPs’ and community pharmacists’ statements were corroborated by observations in the field. The CC’s actual work met a need in the field, and the CC took on non-medical tasks that were nevertheless essential for smooth operation of the process as a whole. This allowed the HCPs to concentrate on tasks related to their medical and/or paramedical expertise. Although the CC had little direct (face-to-face) contact with patients, she nevertheless interacted with them to ensure their ongoing consent and to gather information on changes in their status.

It is noteworthy that overall, the HCPs had a good perception of the CC’s role. The CC’s contribution to communication and transmission of information was greatly appreciated by the HCPs. This might be due (at least in part) by the organisational context in France, where the care of older adults living with frailty is often compartmentalized. There is a lack of coordination between HCPs and between hospital staff and community HCPs, [4,39] which increases the need for care coordination. However, some HCPs did not approve of the CC’s unforeseen involvement in certain tasks. Some community pharmacists questioned the CC’s major involvement in communication tasks. Some GPs questioned the CC’s involvement of CC in the overall coordination of the pathway because they felt that they had lost control of the process. The issue of confidentiality of personal medical data was also raised. The study by De stampa et al. showed similar results, with controversial perceptions of GPs on the place of the care coordinator [22]. Some appreciated not having to carry the additional workload, but some GP felt excluded from the patient case, and felt they were losing supervision of the process. Our results and this previous observation highlight the difficulty to strike a balance for CC between facilitating the communication, without being another person who give information to patients or caregivers.

The present study had several strengths: the combination of a task analysis with semi-structured interviews; the presence of a steering committee throughout the study; and a study protocol that took account of the perceptions of all the various stakeholders (especially the community pharmacists); a three years duration and the interview of 28 HCPs participating to the PAERPA pilot program. Another specific strength was that the CC was not a healthcare professional. We observed positive results, which may support implementation in real setting, while raising issued related to the non-medical background. The study also had several limitations. Firstly, we assessed an integrated care pathway with a medical review initiated in a hospital environment; the population of older adults living with frailty came from a single hospital participating in the French national PAERPA program. Hence, transferability of the findings to other clinical contexts might be limited. Secondly, only one CC had been recruited at the time of the study; hence, some of the HCPs’ observations may be related to the CC’s personal characteristics. Lastly, we did not collect input from patients or caregivers - even though one of the CC’s tasks was to include these individuals in the care pathway. A detailed analysis of the specific tasks performed by GPs and community pharmacists was not performed, although it could have provided interesting and complementary information. It is possible that tasks were transferred by GPs or community pharmacists to the CC because GPs or community pharmacists had additional or unplanned tasks to carry out.

## Conclusion

Our analysis of the CC’s tasks in an integrated care pathway program for frail older patients showed that the program’s overall workload was greater than expected; this difference was primarily due to the CC. In response to needs in the field, the CC became more deeply and extensively involved in three areas: administration, coordination, and communication. These care coordination needs were confirmed by the HCPs included in interviews. Future research should offer a comprehensive task analysis for all stakeholders involved in an integrated care pathway, including CC and HCPs. This would better describe how tasks can be transferred from one stakeholder to another, or how a new set of unanticipated tasks can be transferred between stakeholders. In addition, studies involving a CC without medical background should investigate how the CC strikes a balance between facilitating communication and hindering confidential and interprofessional communication.

## Additional Files

The additional files for this article can be found as follows:

10.5334/ijic.5977.s1 Supplementary Data 1. Consolidated criteria for reporting qualitative studies (COREQ): 32-item checklist.

10.5334/ijic.5977.s2 Supplementary Data 2. Characteristics of the healthcare professionals. Item 16 of the COREQ checklist: domain 2, Settings, Sample description (Study phase 2: sociological surveys).

10.5334/ijic.5977.s3 Supplementary Data 3. Comparative analysis of planned tasks and actual tasks in the medication review process for frail older patients, as implemented at Denain General Hospital.
