# Supplementary material for: The Care Coordinator’s Tasks During the Implementation of an Integrated Care Pathway for Older Patients: A Qualitative Study Based on the French National “Health Pathway of Seniors for Preserved Autonomy” Pilot Program
Source: Int J Integr Care. 2022 Apr 1;22(2):2. doi: 10.5334/ijic.5977 (PMC8973761; doi:10.5334/ijic.5977)
Supplement: Supplementary Data 2. — Characteristics of the healthcare professionals. Item 16 of the COREQ checklist: domain 2, Settings, Sample description (Study phase 2: sociological surveys). [file ijic-22-2-5977-s2.pdf]

Supplementary data 2.

Characteristics of the healthcare professionals. Item 16 of the COREQ checklist: domain 2, Settings, Sample description (Study phase 2: sociological surveys).

Healthcare professional	Age	Sex
GP1	50	M
GP 2	63	M
GP 3	61	M
GP 4	52	M
GP 5	43	F
GP 6	49	M
GP 7	43	F
GP 8	57	M
GP 9	30	M
GP 10	59	M
GP 11	58	F
GP 12	62	M
GP 13	45	F
GP 14	60	M
GP 15	60	M
GP 16	42	M
GP 17	61	M
GP 18	65	M
CPh 1	55	F
CPh 2	33	F
CPh 3	51	F
CPh 4	55	M
CPh 5	30	F
CPh 6	48	F
CPh 7	44	M
CPh 8	59	M
CPh 9	41	F
CPh 10	37	M

GP: general practitioner; CPh: community pharmacist.

Interview / observation duration: Item 21 of COREQ checklist: domain 2, data collection, duration.

Study phase 1: Task Analysis

**HCPs**	**Interview duration**	**Observation duration**
Care coordinator	1 h	27 h 05 min
Nurse	2 h	3 h
Lead investigator (physician)	1 h	/
Administrative coordinator of the integrated care pathway	1 h	/

Study phase 2: sociological surveys

**HCPs**	**Interview duration (min)**
GP 1	23
GP 2	19
GP 3	13
GP 4	21
GP 5	40
GP 6	21
GP 7	23
GP 8	30
GP 9	19
GP 10	22
GP 11	19
GP 12	33
GP 13	8
GP 14	25
GP 15	24
GP 16	17
GP 17	14
GP 18	7
CPh 1	45

CPh 2	31
CPh 3	58
CPh 4	60
CPh 5	37
CPh 6	32
CPh 7	28
CPh 8	26
CPh 9	27
CPh 10	43

HCP: healthcare professional; GP: general practitioner; CPh: community pharmacist.

A non-exhaustive list of verbatim statements by the healthcare professionals about the care coordinator

1) General practitioners (GPs).

GP2: "We don't have time to manage everything [...], so the CC takes some of the load off us".

GP3: "I don’t think that we have to fill out the papers any more,... it's different from the start [of the programme], so that's good [...]; we don’t get annoyed any more because... it's the coordinator who does all the administrative stuff that we had to do".

GP4: "There was something administrative that I was asked about, and I couldn’t give a proper answer. Another person is needed to answer these queries properly...”.

GP6: "The PAERPA process allows tasks to be delegated".

GP7: "The support was valuable because we were discovering how the file was set up, and I think we needed this support”. "Well, the CC is an administrator; she's not from the medical professions, she's not a healthcare professional” and "Physician-patient confidentially is also a problem; the CC is aware of the patient’s confidential medical information".

GP9: " The CC is mainly there to provide information to physicians and other healthcare professionals "; "If it works... in any case, I think that she still has the geriatricians’ support on that... in my opinion... No, I don't see any problem with that".

GP11: "Of course... in all networked or similar systems, it's good when there's a person who really knows the system inside out, with all the tricks of the trade, the phone numbers, the administrative stuff that has to be done, and stuff like that. Otherwise, I reckon it's a little bit overwhelming. It’s complicated"

GP14 "The GP’s role is a bit like that of the pharmacist; you're a stakeholder but you need to know what the others are doing. And there has to be coordinator"; "I am not bothered by the fact that the CC is not a healthcare professional or is not highly qualified; she essentially provides us with administrative support".

GP15: "At the outset, I expected the GP to be at the heart of the project. It ends up being almost everyone except the GP"; "I think the GP should be the coordinator, the referrer. We should do what we did before, and shouldn't be put at the end of the chain - we have the impression that we have been pushed to the end of the chain".

GP16 "(...) it's a pivot, it's a central point that ... it's the first person you might turn to. »

GP18: "[Interviewer]: Would you consider participation in the PAERPA without a CC? - No. She’s the lynchpin of the whole operation”

2) Community pharmacists (CPh)

CPh2 " It [the CC’s work] is essential! She’s the person that links the HCPs together - especially since we do not have any IT support ".

CPh4 ""I think it's good that there's an intermediary because I can't imagine the geriatrician calling us to tell us that so-and-so is about to go home. Maybe I'm wrong but it's not yet common practice. But if there's an intermediary, that's fine with us”.

CPh5 "I think that if she wasn't there to coordinate everything, we would always have to call the GP to give him/her our opinion”.

CPh6 " Well [without the CC], it wouldn't be possible!”

CPh7 " The CC takes on some of the administrative burden"

Ph08 "She intervenes, but afterwards, let's have a quick meeting about this person. Let her stay on to coordinate the project because she does it very well, but, let there be a link that is made by the care coordinator between us.”

CPh9 "It [care coordination] is essential. Otherwise [the process] would fail. "Dialogue with the GPs is not happening... She [the CC] acts as the intermediary”.
