# Supplementary material for: The Care Coordinator’s Tasks During the Implementation of an Integrated Care Pathway for Older Patients: A Qualitative Study Based on the French National “Health Pathway of Seniors for Preserved Autonomy” Pilot Program
Source: Int J Integr Care. 2022 Apr 1;22(2):2. doi: 10.5334/ijic.5977 (PMC8973761; doi:10.5334/ijic.5977)
Supplement: Supplementary Data 3. — Comparative analysis of planned tasks and actual tasks in the medication review process for frail older patients, as implemented at Denain General Hospital. [file ijic-22-2-5977-s3.pdf]

Supplementary Data 3

Comparative analysis of planned tasks and actual tasks in the medication review process for frail older patients, as implemented at Denain General Hospital.

H: hospital staff; HCP: healthcare professional; CC: care coordinator; GP: general practitioner; CPh: community pharmacist; N: nurse; PDP: personal drug plan

		Planned involvement (protocol)				Actual involvement (observed)		
**Step in the process**	**Tasks**	**H**	**HCP**	**CC**		**H**	**HCP**	**CC**
**1. SET-UP**	Patient identification and inclusion	-	-	-		implements	-	-
	Presentation of the PAERPA project	-	receives	gives		-	receives	gives
	Agreement by the HCPs	-	gives	collects		-	gives	checks or collects
	Agreement by the patient	collects	-	-		collects	-	-
	Geriatric assessment of the patient	Implements	-	-		implements	-	-
**2. MEDICATION REVIEW**	Multiple tasks (including creation or transmission of documents for HCPs)	implements	-	-		implements	-	-
**3. INITIATION OF A PDP**	Pre-discharge meeting	-	-	-		implements	-	implements
	Receipt of the documents created and transmitted in step 2 [1]	-	receives	-		-	receives	checks
	Post-discharge coordination with the social services, if required [2]	-	-	-		-	-	implements
	Consultation with the patient	-	implements (GP)	-		-	implements (GP)	-
	Patient-CPh interview 1	-	implements (CPh)	-		-	implements (CPh)	-

Non-modified task   Modified task   New task

		Planned involvement (protocol)				Actual involvement (observed)		
Step in the process	Tasks	H	HCP	CC		H	HCP	CC
	Methodological support	-	requests, if necessary	provides, if necessary		-	always receives (GP)	always provides
**4. LOCAL CLINICAL COORDINATION TEAM**	Validation and completion of data collected at the hospital [3]	-	-	-		-	-	performs
	Summary of all the patient information available at this stage	-	-	-		-	receives (GP)	gives
	Inclusion of other HCPs	-	takes the decision (GP)	-		-	takes the decision (GP)	offers
	Prescription of further assessments	-	takes the decision (GP)	-		-	takes the decision (GP)	-
	Involvement of patients and family [4]	-	-	-		-	-	request
	GP assistance for complex cases	-	requests, if necessary (GP)	follows up, if necessary		-	always receives (GP)	always provides
	Inclusion of other stakeholders	-	takes the decision and contacts the stakeholder (GP)	helps if needed			takes the decision and sometimes contacts the stakeholder (GP)	always contacts the stakeholder
**5. ASSESSMENTS**	Patient-CPh interview 2	-	implements (CPh)	-		-	implements (CPh)	-
	Administrative assistance	-	-	-		-	receives (CPh)	gives
	Multirisk PAERPA assessment, if prescribed	-	implements (N)	-		-	implements (N)	-

Non-modified task Modified task New task

		Planned involvement (protocol)				Actual involvement (observed)		
**Step in the process**	**Tasks**	**H**	**HCP**	**CC**		**H**	**HCP**	**CC**
	Other assessments, if prescribed	-	implements (HCP according to competences)	-		-	implements (HCP according to competences)	-
	Centralization of the assessment results	-	-	-		-	-	implements
	Transmission of the assessment results	-	-	-		-	receives (GP)	Sends
**6. SUMMARY**	Summary of the assessment results	-	implements(face-to-face meeting)	-		-	partly implements (CPh and N, or N alone)	aggregates [5]
	Elaboration of the action plan	-	implements (face-to-face meeting)	-		-	implements (GP)	assists
	Contact with other HCPs and services	-	Implements local clinical coordination team	-		-	implements (GP)	implements
	Administrative assistance	-	-	-		-	receives (GP)	gives
**7. IMPLEMENTATION AND MONITORING**	Launching and monitoring actions	-	implements (GP)	-		-	sometimes implements (GP)	always implements
	Prescribed actions	-	implements (HCP, depending on the skills required)	-		-	implements (HCP, depending on the skills required)	-
	Problem alert	-	-	-		-	-	reports to the GP
	Adaptation of the action plan	-	-	-		-	makes the decision (GP)	-
	Summary of actions carried out	-	-	-		-	receives (GP)	implements
	Close-down	-	decides (GP)	-		-	makes the decision (GP)	-

Non-modified task | Modified task | New task

		Planned involvement (protocol)				Actual involvement (observed)		
Step in the process	Tasks	H	HCP	CC		H	HCP	CC
**8. CLOSE-DOWN**	Reporting logistical problems	-	-	implements		-	-	implements
	Administrative work	-	-	-		-	-	implements

[1] The documents prepared and sent by the hospital stakeholders involved in the PAERPA programme are as follows: a summary of the geriatric assessment results; the medication reconciliation report; the PDP, and a prescription for one week of treatment.

[2] If a problem arose, the care coordinator would be alerted. She would intervene and initiate an urgent response if required, depending on the patient's status at discharge.

[3] The CC entered data about the patient, his/her environment and his/her HCPs when the data were vague or when the hospital had not been able to collect the data due to a lack of time or the lack of a reliable informant. The CC requests non-medical assistance that has not been scheduled.

[4] The CC contacts the patient and the latter's family, in order to (i) confirm their agreement, (ii) involve them in the programme, and (iii) ask them to inform her [the CC] of any changes in the patent's status.

[5] The CC meets with the various HCPs, collects the results of their assessments, and prepares a summary for the GP.

Non-modified task — Modified task — New task
